# Isoform-Specific Role of GSK-3 in High Fat Diet Induced Obesity and Glucose Intolerance

**DOI:** 10.3390/cells11030559

**Published:** 2022-02-05

**Authors:** Manisha Gupte, Sultan Tousif, Jacob J. Lemon, Angelica Toro Cora, Prachi Umbarkar, Hind Lal

**Affiliations:** 1 Department of Biology, Austin Peay State University, Clarksville, TN 37044, USA; jlemon1@my.apsu.edu; 2 Division of Cardiovascular Diseases, The University of Alabama at Birmingham (UAB), Birmingham, AL 35233, USA; tsultan@uabmc.edu (S.T.); torocora@uab.edu (A.T.C.); pumbarkar@uabmc.edu (P.U.)

**Keywords:** GSK-3, high-fat diet, glucose tolerance, obesity

## Abstract

Obesity-associated metabolic disorders are rising to pandemic proportions; hence, there is an urgent need to identify underlying molecular mechanisms. Glycogen synthase kinase-3 (GSK-3) signaling is highly implicated in metabolic diseases. Furthermore, GSK-3 expression and activity are increased in Type 2 diabetes patients. However, the isoform-specific role of GSK-3 in obesity and glucose intolerance is unclear. Pharmacological GSK-3 inhibitors are not isoform-specific, and tissue-specific genetic models are of limited value to predict the clinical outcome of systemic inhibiion. To overcome these limitations, we created novel mouse models of ROSA26CreERT2-driven, tamoxifen-inducible conditional deletion of GSK-3 that allowed us to delete the gene globally in an isoform-specific and temporal manner. Isoform-specific GSK-3 KOs and littermate controls were subjected to a 16-week high-fat diet (HFD) protocol. On an HFD, GSK-3α KO mice had a significantly lower body weight and modest improvement in glucose tolerance compared to their littermate controls. In contrast, GSK-3β-deletion-mediated improved glucose tolerance was evident much earlier in the timeline and extended up to 12 weeks post-HFD. However, this protective effect weakened after chronic HFD (16 weeks) when GSK-3β KO mice had a significantly higher body weight compared to controls. Importantly, GSK-3β KO mice on a control diet maintained significant improvement in glucose tolerance even after 16 weeks. In summary, our novel mouse models allowed us to delineate the isoform-specific role of GSK-3 in obesity and glucose tolerance. From a translational perspective, our findings underscore the importance of maintaining a healthy weight in patients receiving lithium therapy, which is thought to work by GSK-3 inhibition mechanisms.

## 1. Introduction

Obesity has reached pandemic proportions worldwide and is associated with an increased risk of numerous metabolic abnormalities, including insulin resistance, glucose intolerance, and hyperlipidemia, collectively called metabolic syndrome [1,2]. Thus, millions of patients are affected by this condition and at high risk of numerous cardiometabolic diseases, including diabetes, hypertension, atherosclerosis, and heart failure [3,4]. Although the precise mechanism of these devastating metabolic conditions is not fully understood, there is consensus that certain lifestyle factors such as the western diet (high fat and calorie) play a critical role. In addition to food composition, a balance of its intake, need, and storage is crucial for metabolic health.

A protein kinase vital to regulating many aspects of obesity and metabolism is glycogen synthase kinase-3 (GSK-3) [5,6,7,8]. GSK-3 belongs to the serine–threonine kinase family and is ubiquitously expressed [9,10,11]. GSK-3 was initially identified and named based on its ability to inhibit the glycogen synthase, the rate-limiting enzyme of glycogen deposition [11,12]. Since then, 100+ different substrates of GSK-3 have been identified, making GSK-3 among the protein kinases with the most substrates in the cell [11,12]. Unlike most other kinases, GSK-3s are active in resting cells, and various stimuli such as insulin, EGF, and PDGF inactivate it by phosphorylation. In mammals, including humans, GSK-3 exists in two isoforms, GSK-3α (51 kDa) and GSK-3β (47 kDa). GSK-3α/β isoforms are encoded by two distinct genes located on human chromosomes 19 and 3, respectively [9,11]. These isoforms share 98% sequence homology in their catalytic domains but differ significantly in their N and C terminals. However, the overall homology of the two isoforms is 85%; therefore, it is expected that both isoforms share many biological functions. Of note, both isoforms also have unique roles; hence, they cannot compensate for each other. The isoform-specific roles of GSK-3s are clear from the phenotypes in their whole-body KOs [13,14]. Specifically, GSK-3β KO mice are embryonic lethal; however, GSK-3α KO mice survive for several years [15,16].

The function of two key targets of insulin action, glycogen synthase and insulin receptor substrate-1 (IRS1), is suppressed by GSK-3, making it a promising drug target for treatment of insulin resistance and diseases of glucose metabolism (e.g., diabetes) [7,8,17,18,19]. Among the many proposed roles of GSK-3 signaling, the most established are its critical regulatory functions in glucose metabolism, insulin activity, and obesity [17,20]. In agreement with this hypothesis, numerous studies have implicated GSK-3 in the pathogenesis of insulin resistance, metabolic syndrome, and diabetes in both animal and human models [8,21,22,23,24,25]. This hypothesis is further supported by the fact that GSK-3α/β expression and activity are significantly elevated in Type 2 diabetes mellitus (T2DM) patients [5,26,27]. Administration of GSK-3 inhibitors to rodent models of obesity and T2DM have consistently improved insulin sensitivity and glucose homeostasis [8,22,23,28,29]. Together, these studies were crucial to indentifying the role of GSK-3 signaling in glucose metabolism, insulin signaling, and obesity-associated metabolic disorders. However, it is important to note that this work was primarily accomplished by employing one of the following models: (1) pharmacological agent that inhibits both GSK-3 forms non-specifically; (2) genetic models of embryonic global KO; (3) tissue-specific KOs. We and others have demonstrated that there are both distinct roles for GSK-3α and GSK-3β and tissue-specific phenotypes associated with each of these isoforms [30,31,32,33]. Given that chemical inhibitors of GSK-3 cannot discriminate between the two isoforms, it is not feasible to delineate isoform-specific functions of GSK-3s using these drugs. Furthermore, we demonstrated that long-term, nonisoform-specific deletion or inhibition of GSK-3α/β is detrimental [34]. Additionally, genetic embryonic global knockout models are complicated by developmental effects [13,16]. Tissue-specific genetic models are ideal for obtaining cell-specific mechanistic insight; however, these models are of limited value to predict the clinical outcome of systemic inhibition. Thus, none of the models discussed above are comparable or genuine representatives of isoform-specific inhibition in adults. Therefore, it is essential to acquire more knowledge on isoform-specific functions of GSK-3s in the fully mature biological system.

To accomplish this, we created novel mouse models of ROSA26CreERT2-driven, tamoxifen-inducible conditional deletion of GSK-3 that allowed us to delete the gene globally in an isoform-specific and temporal manner, thus avoiding the developmental effects and achieving isoform-specific systemic inhibition. This experimental strategy is comparable to standard chemotherapy with isoform-specific kinase inhibitors in a fully mature system. Therefore, the findings presented herein allow the better prediction of clinical outcomes. Our newly created novel mouse models allowed us to delineate the unique isoform-specific roles of GSK-3 in obesity and glucose clearance. Our findings further supported the hypothesis that GSK-3 isoforms have unique roles in obesity and associated metabolic disorders and are the critical therapeutic target for managing these devastating metabolic disorders.

## 2. Materials and Methods

### 2.1. Generation of Global Conditional GSK-3α and GSK-3β Knockout Mouse Model

To generate conditional GSK-3α global KO (GSK-3α KO) mice or GSK-3β global KO (GSK-3β KO) mice, homozygous GSK-3α flox mice [35,36] or GSK-3β flox mice [34,37] were crossed with mice ubiquitously expressing tamoxifen-inducible Cre recombinase (Cre-ERT2) under the control of the ROSA26 locus (Jackson Lab #008463, ROSA26CreERT2) [38]. This crossing resulted in heterozygous mice for both flox and Cre alleles. These heterozygotes were backcrossed with respective flox/flox animals to generate flox/flox-Cre (KOs) alleles. Littermate flox/flox animals (Cre-negative) were employed as controls. All of the mouse strains had the C57BL/6 background. At 3 months of age, all of the mice were subjected to a tamoxifen diet for 2 weeks (Teklad Diet. 130860, Envigo, Madison, WI, USA) followed by four weeks of washout to eliminate any residual tamoxifen or tamoxifen-induced toxicity. The tamoxifen diet protocol has been well-established in our laboratory and consistently reported [21,36]. Baseline parameters such as body weight, fat mass, lean mass, glucose tolerance, and insulin tolerance were assessed in all of the animals. Following the baseline analyses, WT (n = 5–8 per group) and KO (n = 5–11 per group) animals were fed either a control/chow diet (CD) or high-fat diet (HFD) for 16 weeks. The Institutional Animal Care and Use Committee of Vanderbilt University Medical Center approved all animal procedures and treatments (protocol # M1700133-00). All animals were housed in a temperature-controlled room with a 12:12 h light/dark cycle and received humane care.

### 2.2. Mice

Genotypes of the conditional knock-out and control mice were as follows:

GSK3α (fl+/+)/ROSA26-ER-CRE (−/−) Tamoxifen, control (littermate control to GSK-3α KO);

GSK3α (fl+/+)/ROSA26-ER-CRE (+/−) Tamoxifen, GSK-3α KO;

GSK3β (fl+/+)/ROSA26-ER-CRE (−/−) Tamoxifen, control (littermate control to GSK-3β KO);

GSK3β (fl+/+))/ROSA26-ER-CRE (+/−) Tamoxifen, GSK-3β KO.

### 2.3. Diet

After baseline measurements, GSK-3α KO, GSK-3β KO, and littermate control mice were subjected to a high-fat diet (HFD) for 16 weeks. The HFDs consisted of 20% protein, 20% carbohydrates, and 60% fat (Research diet Inc. Cat # D12492, New Brunswick, NJ, USA), which provided 5.24 (HFD) kcal/g. Diets were provided ad libitum to mice. Additionally, to investigate the effect of GSK-3β deletion on a normal diet, WT or GSK-3βKO mice were fed a chow diet (Research diet Inc. Cat # D12450K) for 16 weeks.

### 2.4. Body Composition

Lean and fat mass were measured in conscious mice at baseline, 4, 8, 12, and 16 weeks post-HFD using the Minispec Model mq7.5 (Bruker Instruments).

### 2.5. Oral Glucose Tolerance Test (GTT)

An oral glucose tolerance test was performed after 6 h of fasting. The first glucose measurement was recorded at the end of 6 h prior to glucose administration (0 time point). After administration of glucose orally at the dose of 2 g/kg body weight, subsequent blood glucose measurements were taken at 15, 30, 45, 60, 90, and 120 min using a glucometer (Freedom Freestyle Lite; Abbott laboratories).

### 2.6. Insulin Tolerance Test (ITT)

Standard human insulin (Novo Nordisk, Princeton, NJ, USA) was injected intraperitoneally (O.5 IU/kg body weight). The first glucose measurement was recorded at the end of 6 h prior to insulin administration (0-time point). After the administration of insulin intraperitoneally, blood glucose measurements were taken at 15, 30, 45, 60, 90, and 120 min using a glucometer.

### 2.7. Statistical Analysis

Data are expressed as mean ± SEM. Differences between the two groups were analyzed using an unpaired *t*-test (Graph Pad Prism Software Inc., San Diego, CA, USA). Significance was accepted at *p* < 0.05.

## 3. Results

### 3.1. Creation and Characterization of Global Conditional GSK-3α Knock-Out (KO) Mice

**Figure 1:**
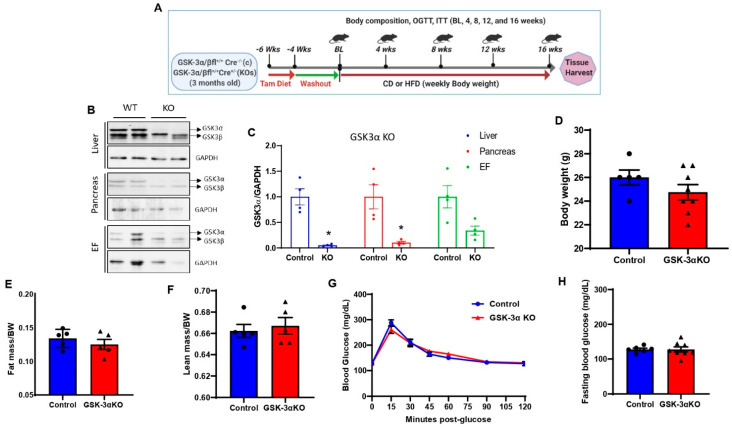
Creation and characterization of global conditional GSK-3α KO mouse model. (**A**) Experimental design, three-month-old male controls, or GSK-3 KO mice were subjected to a tamoxifen diet for 2 weeks followed by 4 weeks of washout. Following the washout period, control and GSK-3 KO mice were fed a CD and an HFD, respectively, for sixteen weeks. (**B**) Representative immunoblot showing the specific deletion of GSK-3α in the liver, pancreas, and epididymal fat in GSK-3α KO mice at the termination of the experiment (16 weeks post chow/HFD treatment). The replicates of different samples were analyzed. (**C**) Quantification of GSK-3α expression from control and GSK-3α KO tissues showed a significant reduction in GSK-3α expression in KO compared to controls. Baseline body weight (**D**), fat mass (**E**), lean mass (**F**), glucose tolerance test (**G**), and fasting blood glucose (**H**) in controls and GSK-3α KO mice; * *p* < 0.05 WT vs. KO.

Global inducible GSK-3α or GSK-3β KO mice were generated by crossing GSK-3α flox mice or GSK-3β flox mice with ubiquitously expressing tamoxifen-inducible Cre recombinase (Cre-ERT2) under the control of the ROSA26 locus. At 3 months of age, animals were subjected to a tamoxifen diet for 2 weeks (Teklad Diet. 130860) followed by 4 weeks of washout to eliminate any residual tamoxifen or tamoxifen-induced toxicity (Figure 1A). The deletion efficiency was determined at the termination of the experiment (16 weeks post chow/HFD treatment; Figure 1A,B). Tamoxifen treatment significantly reduced the expression of GSK-3α in metabolic tissues such as the liver, pancreas, and adipose tissue (EF) in GSK-3α KO mice compared to littermate controls (Figure 1B,C). At baseline, there were no significant differences in body weight, fat mass, or lean mass between controls and GSK-3αKO mice (Figure 1D–F). Since previous studies in germline GSK-3α KO mice exhibited improved glucose clearance [35,39], we tested glucose clearance and insulin sensitivity in controls and conditional GSK-3α KO mice. At baseline, controls and conditional GSK-3α KO showed comparable glucose clearance (Figure 1G) and insulin sensitivity (data not shown). Additionally, fasting blood glucose levels were unaltered between controls and conditional GSK-3α KO mice (Figure 1H).

### 3.2. Conditional Global GSK-3α Deletion Protects from HFD-Induced Obesity but Plays a Minimal Role in Glucose Clearance

**Figure 2:**
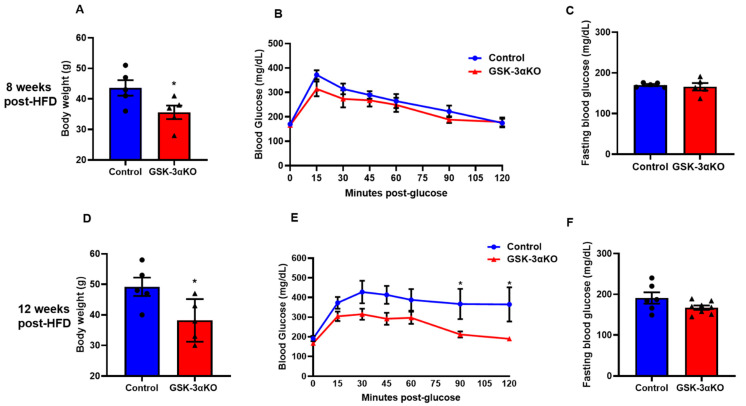
Conditional global GSK-3α deletion protects the mice from HFD-induced obesity but has a minimal role in glucose clearance. GSK-3α KO and control animals were subjected to an HFD and were analyzed at 8 and 12 weeks post-HFD. Body weight (**A**), glucose tolerance test (**B**), and fasting blood glucose (**C**) in controls and GSK-3α KO animals after 8 weeks on an HFD. Body weights (**D**), Glucose tolerance test (**E**), and fasting blood glucose (**F**) in controls and GSK-3α KO animals after 12 weeks on an HFD; * *p* < 0.05 WT vs. KO.

Littermate controls and conditional GSK-3α KO mice were subjected to an HFD to induce obesity, and body weight and glucose clearances were observed at 4, 8, 12, and 16 weeks. Interestingly, HFD-induced body weight gain was significantly less in conditional GSK-3α KO mice compared to their littermate controls (Figure 2A,D). We did not see any difference in fasting blood glucose levels between the HFD-fed controls and conditional GSK-3αKO mice post-HFD (Figure 2C,F). Interestingly, the blood glucose clearance curves of GSK-3α KO mice and controls started to separate at 8 weeks post-HFD and the difference reached statistical significance at 12 weeks, demonstrating a significantly improved clearance in GSK-3α KOs (Figure 2B,E). GSK-3α KOs continued to show improved glucose clearance and significantly lower body weight at 16 weeks post-HFD (Supplement Figure S1). However, as body weight is a critical determinant of glucose clearance, the significantly lower body weights of GSK-3α KOs may be contributing to their improved glucose clearance phenotype. We adjusted the glucose load for the GTT assay as per the body weight of the individual animal in all groups. Taken together, these results indicate that GSK-3α deletion modestly improves glucose clearance.

### 3.3. Baseline Characteristics of Conditional GSK-3β KOs

**Figure 3:**
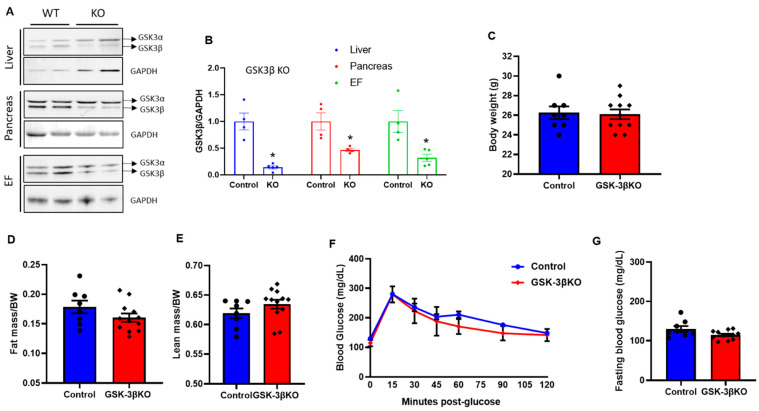
Creation and characterization of global conditional GSK-3β KO mouse model. (**A**) Representative immunoblot showing specific deletion of GSK-3β in the liver, pancreas, and epididymal fat in GSK-3βKO mice. (**B**) Quantification of GSK-3β expression from GSK-3β KO and control tissues showing a significant reduction in GSK-3β expression in KO mice compared to controls at the termination of the experiment (16 weeks post chow/HFD treatment). The replicates of different samples were analyzed. Baseline body weight (**C**), fat mass (**D**), lean mass (**E**), glucose tolerance test (**F**), and fasting blood glucose (**G**) in controls and GSK-3β KO mice; * *p* < 0.05 WT vs. KO.

The tamoxifen treatment and washout protocol were consistent as described for GSK-3α KOs and depicted in Figure 1A. Tissues from global conditional GSK-3β KO mice were analyzed and compared to those of controls using immunoblotting and protein quantification to determine GSK-3β expression in the liver, pancreas, and adipose tissue. GSK-3β protein expression levels in KO mice were significantly lower in all three tissue types compared to those their littermate controls (Figure 3A,B). At baseline, there were no significant differences in body weight, fat mass, or lean mass between conditional GSK-3β KOs and littermate controls (Figure 3C–E). At baseline, there was a trend of slight improvement in glucose clearance and fasting blood glucose levels in KOs compared to controls; however, this did not reach statistical significance (Figure 3F,G). Notably, there was no phenotype in GSK-3α KOs at baseline; therefore, even slight (though insignificant) improvement in glucose clearance and fasting blood glucose levels in GSK-3β KOs at baseline indicated GSK-3β as a comparatively dominant isoform in glucose metabolism.

### 3.4. Conditional Global GSK-3β Deletion Protects from HFD-Induced Glucose Intolerance

**Figure 4:**
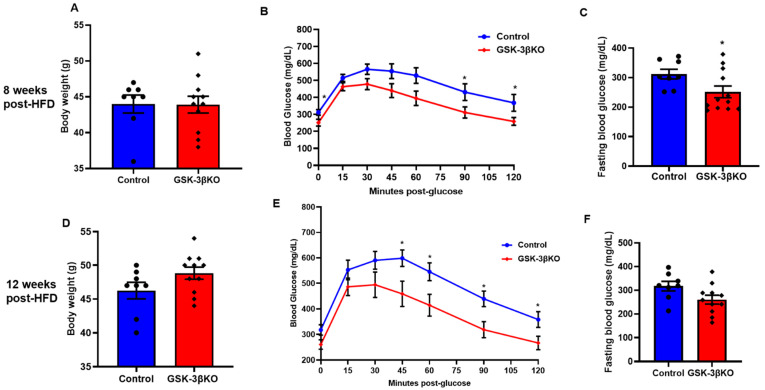
Conditional global GSK-3β deletion protects GSK-3β KO mice from HFD-induced glucose intolerance. GSK-3β KO and control animals were subjected to an HFD and were analyzed at 8 and 12 weeks post-HFD. Compared to the controls, GSK-3β KO mice exhibited improved glucose clearance at both timepoints. (**A**) Body weight, (**B**) glucose tolerance test, and (**C**) fasting blood glucose in GSK-3β KO and control animals after 8 weeks on an HFD. (**D**) Body weight, (**E**) glucose tolerance test (GTT), and (**F**) fasting blood glucose in controls and KO animals after 12 weeks on an HFD; * *p* < 0.05 WT vs. KO.

**Figure 5:**
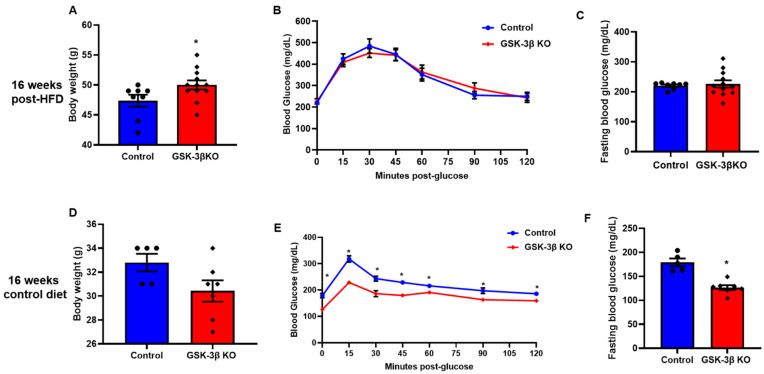
GSK-3β deletion mediated improved glucose clearance is dependent on body weight maintenance. (**A**) After 16 weeks on an HFD, GSK-3β KO mice had a significantly higher body weight compared to their littermate controls. After 16 weeks on an HFD, GSK-3β KO mice exhibited comparable glucose clearance and fasting blood glucose to their littermate controls on the same diet (**B**,**C**). (**D**–**F**) Notably, after 16 weeks on the control diet, GSK-3β deletion improved glucose clearance and fasting blood glucose in KO mice despite comparable body weights; * *p* < 0.05 WT vs. KO.

Littermate controls and conditional GSK-3β KO mice were subjected to an HFD to induce obesity. Body weight and glucose clearances were observed at 4, 8, 12, and 16 weeks. Up to 4 weeks post-HFD treatment, fasting blood glucose levels, body weight, and blood glucose clearance were comparable in conditional GSK-3β KO and littermate controls (data not shown). However, at 8 weeks post-HFD feeding, GTT assay revealed a significantly improved glucose clearance in conditional GSK-3β KO mice compared to controls (Figure 4B). Remarkably, the body weight of GSK-3β KO mice and controls was comparable, suggesting that observed improvement in glucose clearance (GTT assay) is not confounded by body weight and is indeed a metabolic response of loss of GSK-3β (Figure 4A). Of note, an ITT assay revealed a comparable insulin sensitivity in GSK-3β KO mice and littermate controls (data not shown). Thus, enhanced insulin sensitivity did not facilitate improved glucose clearance in GSK-3β KOs. At 12 weeks post-HFD, GSK-3βKO mice continued to show improved glucose clearance despite a trend of increased body weight gain in the KO group (Figure 4D,E). Consistently, fasting blood glucose was lower in GSK-3β KOs compared to controls at both time points (Figure 4C,F). However, the GSK-3βKOs on chronic HFD (16 weeks) showed a significant increase in body weight compared to littermate controls (Figure 5A). Furthermore, comparable GTT and fasting blood glucose levels suggest that the metabolic benefits in GSK-3β KOs were lost at 16 weeks post-HFD (Figure 5B,C). The increased body weight in KOs most likely accounts for the loss of metabolic benefits of GSK-3β deletion. Optimistically, conditional GSK-3β KOs maintained on control diet continued to demonstrate comparable body weight and metabolic benefits, as reflected by improved glucose tolerance and significantly lower fasting blood glucose (Figure 5D–F). Taken together, these results suggest that among the GSK-3 isoforms, GSK-3β is the dominant regulator of glucose metabolism and its inhibition leads to significant metabolic benefit. However, its chronic inhibition may lead to increased body weight (obesity); therefore, strategies to maintain the body weight are critical to sustaining the metabolic benefit of GSK-3β inhibition.

## 4. Discussion

We created novel mouse models of inducible deletion of GSK-3 to achieve global and isoform-specific conditional deletion at desired time points. These novel genetic models allowed us to determine the metabolic outcome of isoform-specific deletion of GSK-3s globally. This was not feasible with previously reported genetic models as GSK-3α global KO exhibits several developmental defects [13,16], and GSK-3β KO is embryonic lethal [15]. Our findings suggest that although deletion of either GSK-3 isoform protects against HFD-induced glucose intolerance, GSK-3β is clearly the dominant isoform regarding GSK-3 effects on glucose metabolism. Interestingly, the effect of GSK-3 isoform deletion on HFD-induced obesity was completely the opposite: GSK-3α KOs were protected; however, GSK-3β KOs on chronic HFDs gained more weight, thereby losing the protective effect on glucose tolerance. GSK-3β KOs on the control diet maintained the improved glucose clearance with comparable body weight to the controls.

GSK-3α KOs were protected against HFD-induced obesity and glucose intolerance. Although the literature on the influence of GSK-3α on metabolism is limited compared to the GSK-3β isoform, there are two contradictory reports from the renowned Woodgett and colleagues [35,39]. In an elegant paper, MacAulay et al. [35] demonstrated that global embryonic GSK-3α knockouts display improved glucose tolerance, insulin sensitivity, and significantly lower fat mass. However, the same group later failed to reproduce these findings in an inbred strain of C57BL/6 animals and tissue-specific KOs of skeletal muscle and liver [39]. Since the original study was conducted with an outbred strain of mice (ICR), the authors concluded that the protective effect of GSK-3α deletion is mouse-strain-dependent. In complete disagreement with this hypothesis, our study of inbred C57BL/6J-GSK-3α KOs displayed significant protection from HFD-induced obesity and glucose intolerance. Consistent with Patel et al. [39], we did not observe improved GTT, ITT, or lower fat mass in GSK-3α-KOs at baseline (control diet group). Of note, both previous studies lacked the HFD group; therefore, how these previously reported GSK-3α KO models would behave once subjected to an HFD is unknown. Patel et al. [39] also employed tissue-specific models of skeletal muscle and liver-specific GSK-3α KOs. The absence of a metabolic phenotype in these tissue-specific models suggests that the phenotype in the global model depends on tissues other than or in addition to skeletal muscle and liver. The mechanism and tissues responsible for the improved glucose tolerance in our GSK-3α KOs are currently unknown and require further investigation. McCamphill et al. [40] recently developed a set of GSK3 isoform-selective inhibitors; the GSK3α specific inhibitor BRD0705 sufficiently corrected fragile X syndrome and displayed great potential in treating acute myeloid leukemia (AML) [40,41]. Importantly, these benefits were seen without side effects such as those associated with other GSK-3 inhibitors in clinical development. Our findings provide encouragement to test the efficacy of this newly developed isoform-specific GSK-3α inhibitor against obesity, glucose clearance, and other metabolic conditions.

In addition to glucose tolerance, MacAulay et al. [35] also reported significantly improved insulin sensitivity in the global embryonic GSK-3α KOs at baseline. In stark contrast, we did not observe enhanced insulin sensitivity in our study with either diet regime, control, or HFD. Although it is difficult to reconcile these findings, we think that the employed animal models in various studies may account for these variable results. Of note, the global GSK-3α KO used by MacAulay et al. [35] was an embryonic deletion model that has been reported for several developmental defects [13,16]. Additionally, compensatory mechanisms are well-known confounders with the KO model of embryonic deletion. Numerous studies with various tissue-specific KOs of GSK-3β and pan-pharmacological inhibitors have shown improved insulin sensitivity, increased survival, and even regeneration of the β-cell mass [23,42,43,44,45]. Considering these reports, we hypothesized that enhanced glucose clearance in our GSK-3β KOs was facilitated by improved insulin sensitivity. However, surprisingly, the insulin sensitivity was comparable in GSK-3β-KO and littermate controls (data not shown). These findings suggest that the observed enhanced glucose clearance in our GSK-3α KO or GSK-3β KOs was facilitated by the mechanisms independent of insulin sensitivity.

The GSK-3β KOs displayed improved glucose clearance and gained more weight on an HFD than their littermate controls. Interestingly, the increased body weight and improved glucose clearance phenotype are also seen in humans on chronic lithium therapy, the only GSK-3 inhibitor in clinical use [46,47,48]. The administration of lithium to psychiatric patients lowered blood glucose levels and improved glucose tolerance [49,50]. Moreover, lithium showed a hypoglycemic effect in diabetes patients. Consistently, lithium has reduced blood glucose levels in mouse models of diabetes [51,52]. Furthermore, increased body weight gain is a well-known side effect of lithium therapy [53,54,55]. To the best of our knowledge, none of the previously reported genetic models of GSK-3 isoforms could mimic both these key phenotypes as observed in humans. GSK-3 isoforms express ubiquitously, and tissue-specific models have displayed the cell-specific role of GSK-3 isoforms at various aspects of metabolism. Therefore, the observed unique phenotype in these global conditional KOs is not entirely unexpected. We speculate that the observed metabolic phenotype is potentially the outcome of multiple organs’ contribution and crosstalk; thus, simultaneous deletion in all metabolic tissues facilitated this translationally relevant phenotype. It is important to note that in addition to a modest inhibition of GSK-3, lithium works through various additional mechanisms; therefore, caution should be taken when comparing our findings with lithium effects in humans. Nonetheless, the global GSK-3β conditional KO model described here will be invaluable for future studies to delineate the underlying mechanism of these clinically relevant phenotypes. These future studies may help understand the mechanism of increased body weight gain in patients on lithium therapy. Interestingly, GSK-3β KOs maintained on the control diet for several months could retain comparable body weight to their littermate controls and had improved glucose clearance. These findings suggest that maintaining a healthy weight is of prime importance for patients on chronic lithium therapy.

Finally, although we think that our genetic models are optimal translational preclinical experimental settings, we recognize a few limitations and critical areas of improvement. These include the developmental obesity model (gene is deleted before the onset of obesity) and the magnitude of inhibition. The absolute genetic deletion is different from the level of inhibition seen with pharmacological agents, e.g., the therapeutic range of lithium is 0.5 to1.0 mM, which leads to ~20–30% inhibition of GSK-3. Therefore, future studies with heterozygous animals (~50% deletion) and an established obesity model (gene deletion after obesity is established) are warranted.

## 5. Conclusions

Our novel mouse models allowed us to delineate the isoform-specific role of GSK-3 in obesity and glucose clearance. Our findings restate the importance of developing and testing highly selective GSK3 isoform-specific inhibitors for optimal metabolic benefits and avoiding off-target effects of nonisoform-specific inhibitors. From a translational perspective, our findings underscore the importance of maintaining a healthy weight for patients on lithium therapy, which is thought to work by GSK-3 inhibition mechanisms. Our findings support the hypothesis that GSK-3 isoforms have unique roles in obesity and associated metabolic disorders and are the critical therapeutic target to manage these devastating metabolic disorders.

## Data Availability

Not applicable.

## Supplementary Materials

The following are available online at https://www.mdpi.com/article/10.3390/cells11030559/s1, Figure S1. Body weights, glucose tolerance test, and fasting blood glucose in 16 weeks HFD-fed Control and GSK-3α KO mice.
