# Supplementary material for: Isoform-Specific Role of GSK-3 in High Fat Diet Induced Obesity and Glucose Intolerance
Source: Cells. 2022 Feb 5;11(3):559. doi: 10.3390/cells11030559 (PMC8834358; doi:10.3390/cells11030559)
Supplement: Supplementary file 1 [file cells-11-00559-s001.zip › cells-1537860-supplementary.pdf]

Supplementary Material

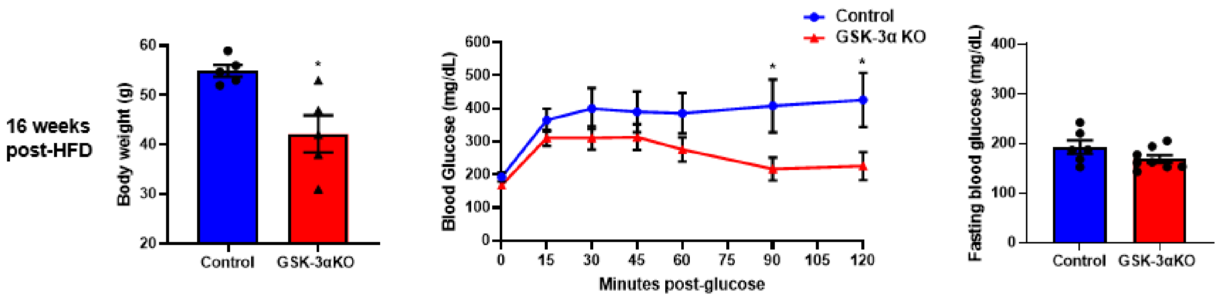

Supplement Figure S1: Chronic conditional global GSK-3α deletion protect from HFD-induced glucose intolerance : GSK-3α KO and control animals were subjected to HFD diet and were analyzed at 16 weeks post-HFD. Compared to the controls, GSK-3α KO mice exhibited improved glucose clearance. (**A**) Body weights, (**B**) glucose tolerance test, (**C**) fasting blood glucose in GSK-3α KO and control animals; * $p < 0.05$ WT vs KO.
